# Super pulsed thulium fiber laser outcomes in retrograde intrarenal surgery for ureteral and renal stones: a systematic review and meta-analysis

**DOI:** 10.1186/s12894-023-01355-x

**Published:** 2023-11-07

**Authors:** Nazal A. Almasoud, Omar Safar, Adel Elatreisy, Saad Thamer Alshahrani, Saud Bin Libdah, Sulaiman M. Alkhaldi, Nezar F. Alsoliman, Abdulrahman M. Alderaan, Ibrahim Abdel-Al, Tamer A. Abouelgreed, Mohammed Alabeedi, Abdulrahman Al-Aown

**Affiliations:** 1 Urology Department, Prince Mutaib Bin Abdulaziz Hospital, Sakaka, Aljouf province Saudi Arabia; 2 Urology Department, Armed Forces Hospital Southern Region, Khamis Mushayt, Aseer Province Saudi Arabia; 3 Urology Department, Faculty of Medicine, Al-Azher University, Cairo, Egypt; 4 Urology Department, Faculty of Medicine, Al-Azher University, Assiut Branch, Assiut, Egypt

**Keywords:** Lithotripsy, Laser, Thulium, Kidney stone

## Abstract

**Background:**

Laser lithotripsy using a thulium fiber laser (TFL) has become an effective treatment option for small renal stones with low complication rates. TFL has a higher absorption coefficient, smaller fibers, and better pulse rate capability.

**Methods:**

We conducted a systematic review and meta-analysis to evaluate the published evidence regarding TFL's lithotripsy performance in retrograde intrarenal surgery (RIRS), for which we primarily assessed the outcomes of stone-free rate, operation time, and complications. We searched different databases from inception to April 2023. We assessed the methodological quality and risk of bias using the Cochrane Risk of Bias tool for randomized trials and the ROBINS-I tool for non-randomized studies. We used a random-effects model for meta-analysis and assessed heterogeneity using the I2 statistic.

**Results:**

Twelve published studies evaluated the efficacy of RIRS using a TFL for treating renal and ureteral stones. The meta-analysis revealed a predicted stone-free rate of 89.37% (95% CI: 83.93% to 93.12%), indicating that, on average, approximately 89.37% of patients achieved a stone-free state after treatment. The substantial heterogeneity among the studies was evident, as shown by a Q-value of 33.1174 and a *p*-value of 0.0003. The I^2^ value of 69.80% (95% CI: 25.91% to 92.02%) highlighted the proportion of variability attributed to genuine heterogeneity across the studies. Moreover, the H^2^ value 3.31 (95% CI: 1.35 to 12.53) indicated significant heterogeneity beyond random chance. The estimated overall effect size (logit-transformed) of 2.1289 was highly statistically significant (z = 8.7648, *p* < 0.0001) with a confidence interval of 1.6528 to 2.6049. The reported complications varied across studies, encompassing Clavien grade I–II complications in most cases, with a subset experiencing more severe Clavien grade III–V complications. Additionally, other studies noted a range of complications, such as haematuria, fever, transient creatinine elevation, and postoperative issues like bleeding, pain, and sepsis.

**Conclusion:**

This meta-analysis suggests that RIRS using TFL is an effective and safe treatment option for renal and ureteral stones, with high stone-free and low complication rates. The included studies exhibited a low risk of bias and were of high quality. However, more extensive randomized controlled trials with extended follow-up periods are needed to investigate this technique's efficacy and safety.

## Introduction

Renal and ureteral stones are among the most frequent disorders, resulting in patient misery, lost labour, and morbidity. Urolithiasis is prevalent in 2.8% of Americans and 1.5% of Europeans. In addition, the high chance of recurrence associated with urinary tract disease has been observed to be about 50% within ten years [1]. This problem has plagued humans since ancient times, with evidence dating back to 4000 B.C. [2]. Over the past two decades, flexible ureteroscopes have significantly improved the safe and effective treatment of small kidney stones (2 cm) with high success rates and minimal patient discomfort [3]. Laser lithotripsy has become the primary treatment option for renal calculi due to its established efficacy [4, 5]. Retrograde intrarenal surgery (RIRS) is a minimally invasive surgical technique that can be used to treat kidney stones. The super-pulsed thulium fiber laser is used in RIRS to disintegrate/break stones into tiny particles [4, 5]. The thulium fiber laser (TFL) is a safe and efficient lithotripsy method with low complication rates [6].

Recent developments in laser fiber technology have been mainly responsible for the evolution of a new generation of lasers. Compared to Holmium:yttrium–aluminium-garnet (Ho: YAG), it has been found to have a four times greater absorption coefficient in water-containing tissue [7]. One of this laser's key benefits is the fiber's miniaturization. Because it makes the use of smaller operating fibers (< 200 μm core diameter) a possibility [8], it also generates lower energy pulses (0.025 Joules or J) and better pulse rate capability (up to 2 kHz).

The procedure involves the insertion of a flexible ureteroscope through the urethra and into the renal collecting system. Once the stone is visualized, laser energy is used to fragment the stone into smaller pieces, which are then removed using a basket or suction. Super-pulsed TFL operates at a wavelength of 1.94 μm and emits short pulses of laser energy at low peak powers [9]. TFL allows the fragmentation of stones without causing thermal damage to the surrounding tissue. In addition, the short pulse duration reduces the risk of stone retropulsion and improves the stone fragmentation efficiency.

Research studies have investigated the safety and efficacy of RIRS using super-pulsed TFL for managing stones. However, there has yet to be a systematic review and meta-analysis of the available literature. This review aims to address this gap in the literature by synthesizing the available evidence on the safety and efficacy of RIRS using super-pulsed TFL. Furthermore, this systematic review and meta-analysis provide a comprehensive assessment of the safety and efficacy of this technique, which helps inform clinical practice and guide future research in this field. Since it is an emerging and novel technique we would additionally compare the findings of this study with the standard Ho: Yag laser technique and highlight the comparative analysis or discussion in terms of efficacy, technique and outcome.

## Methods

The Preferred Reporting Items for Systematic Reviews and Meta-Analyses (PRISMA) statement was followed when reporting this systematic review and meta-analysis. The Prospero registration number was given to the protocol of this systematic review [CRD42023432214].

### Study outcomes

The primary outcome, which serves as the central focus of our investigation, is the stone-free rate. This measure reflects the success of the intervention in effectively eliminating renal stones. As the cornerstone of our analysis, the stone-free rate directly assesses the efficacy of the treatment modality under study. Stone-free rate definitions varied among the studies. For instance, some studies defined stone-free rate as the absence of residual fragments larger than 2 mm, whereas others considered any size of residual fragments or used a threshold of 2 mm. Regrettably, a few studies did not provide specific criteria for SFR.

In contrast, secondary outcomes encompass a range of variables, including operation time, complications, laser time, and ablation speed. While these secondary outcomes contribute valuable insights into various facets of the intervention, they are of secondary importance to the primary objective of evaluating the stone-free rate. As such, these secondary outcomes are analyzed and reported to offer a comprehensive overview of the intervention's broader effects.

### Search strategy and study selection

Two investigators independently searched PubMed, Google Scholar, Science Direct, Clinicaltrial.gov, and Cochrane library databases from inception to August 2023 to identify studies evaluating the efficacy of RIRS using TFL for treating renal stones. The electronic search strategy used the keywords 'retrograde intrarenal surgery,' 'RIRS,' 'super pulsed thulium fiber laser,' 'TFL,' 'renal stones,' and 'kidney stones.' Additionally, the reference lists of screened full-text studies were checked for other potentially eligible studies. To determine eligible studies, inclusive selection criteria were applied. These criteria required that the study population consist of patients with renal stones/ ureteral stones undergoing RIRS with TFL, and studies should report stone-free rates, operation time, and complications. Studies were excluded if they did not assess the outcomes of interest or were observational studies without our required outcome measures, case series, case reports, or animal studies. The most comprehensive publication was used if several studies involved the same population. Any discrepancies were resolved through discussion and adjudication by a senior reviewer. All the included studies were peer-reviewed and published.

### Screening and data extraction

In the first phase of study selection, articles with irrelevant titles were excluded. Subsequently, in the second phase, abstracts and full texts of articles were reviewed to include those matching the inclusion criteria. Endnote X8 was utilized to organize and assess titles and abstracts and identify duplicate entries. A double screening technique was employed to ensure high-quality results, with one evaluation for titles and abstracts and the other for full texts. A piloted data-extraction sheet was used to gather information regarding the study period, study design, sample size, study region, stone location, and patient age. The study's predetermined outcomes were stone-free rates, operation time, and complications. Two investigators performed data extraction independently, and any discrepancies were resolved by consensus without simplifying or making assumptions.

### Quality assessment in individual studies

The ROBINS-I tool for non-randomized studies assessed the methodological quality and potential risk of bias in non-randomized clinical studies. This tool assesses the risk of bias in the following domains: confounding, selection of participants, classification of interventions, deviation from interventions, missing data, measurement of outcomes, and selection of reported results.

### Statistical analysis

A random-effects model was used regardless of heterogeneity, and the I^2^ statistic was used to report heterogeneity. I^2^ > 50% indicates significant heterogeneity. Results were considered statistically significant at *P* < 0.05. All statistical analyses were conducted using R software.

Additionally, we assessed publication bias, which is vital for evaluating the credibility and reliability of our meta-analysis results. Funnel plots were employed to visualize potential publication bias.

## Results

### Search results

**Fig. 1:**
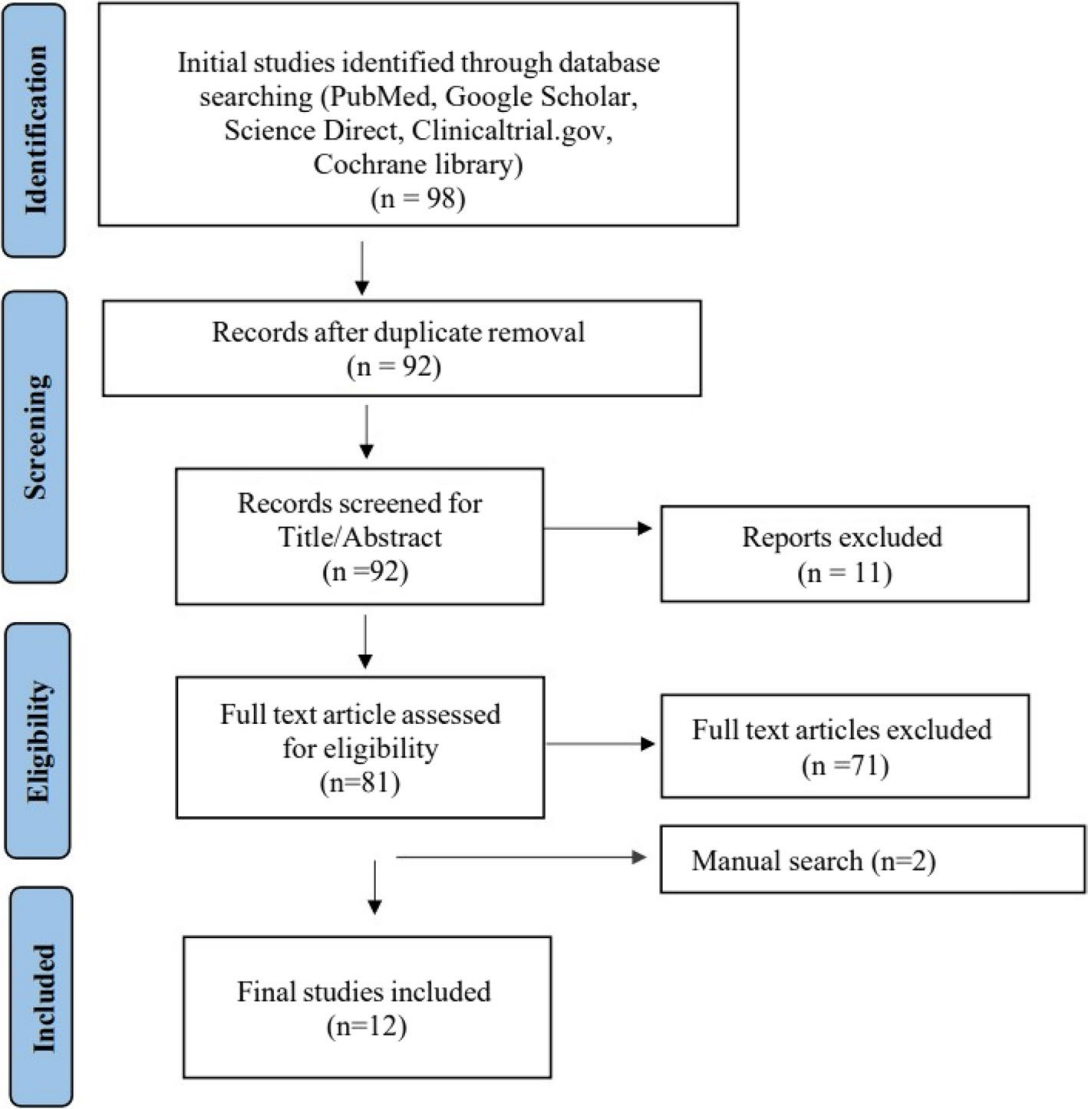
PRISMA Flow chart showing study selection

The search strategy identified 92 publications, which were screened for relevance. Upon reviewing the full text of relevant abstracts, 80 articles were assessed, of which only 10 met all the inclusion criteria and were selected for the review. We also found two more studies through manual searches, which were included in the final analysis, resulting in 12 studies. The search and selection processes are depicted in Fig. 1 with a detailed flowchart.

### Results of quality assessment

As per Cochrane Robbins I, all included studies exhibited a low risk of bias. A rating of 'Low' typically indicates a low risk of bias for each evaluation criterion. This suggests that the studies being assessed have taken measures to minimize potential biases and methodological flaws in areas such as confounding, participant selection, intervention classification, deviation from intervention, missing data handling, outcome measurement, and reporting of results, as indicated by the quality assessment. All of the included studies showed satisfactory results, as presented in Table 1.

**Table 1:** Quality assessment

Studies	Confounding	Selection of participants	Classification of interventions	Deviation from intervention	Missing data	Measurement of outcomes	Selection of reported results	Overall
Delbarre et al. [10]	Low	Low	Low	Low	Unclear	Low	Low	Low
Singh A et al. [11]	Low	Low	Low	Low	Unclear	Low	Low	Low
Quiroz Madarriaga Y. et al. [12]	Low	Low	Low	Low	Unclear	Low	Low	Low
Geavlete B. et al. [13]	Low	Low	Low	Low	Unclear	Low	Low	Low
Soundarya G. et al. [14]	Low	Low	Low	Low	Unclear	Low	Low	Low
Sytnik D. et al. [15]	Low	Low	Low	Low	Unclear	Low	Low	Low
Taraktin M. et al. [4]	Low	Low	Low	Low	Unclear	Low	Low	Low
Vaddi C.M. et al. [16]	Low	Low	Low	Low	Unclear	Low	Low	Low
Sierra et al. [17]	Low	Low	Low	Low	Unclear	Low	Low	Low
Taraktin M. et al. [18]	Low	Low	Low	Low	Unclear	Low	Low	Low
Enikeev D. et al. [19]	Low	Low	Low	Low	Unclear	Low	Low	Low
Korolev D. et al. [20]	Low	Low	Low	Low	Unclear	Low	Low	Low

### Characteristics of the studies included

The characteristics of the included studies are summarized in Table 2. The selected studies were published between 2020 and 2023, with the mean age of participants ranging from 8.5 to 60.1 years, and the follow-up periods predominantly spanned three months across most studies. These studies were conducted across various regions, including Europe, Asia, the US, and India. The research designs varied and included prospective clinical trials, retrospective studies, and clinical studies [4, 10–20]. Notably, most studies included a comparison arm, except three studies, as outlined in Table 2.

**Table 2:** Baseline characteristics of the studies included in this review

Studies	Country	Study design	Year of publication	Baseline comparison (Y/N)	Number of participants (TFL)	Mean age (years)	Follow-up
Delbarre et al. [10]	France	Prospective study	2023	Yes	100	60.1 ± 17.7	3 months
Singh A et al. [11]	India	Prospective clinical trial	2023	NR	76	NR	NR
Quiroz Madarriaga Y. et al. [12]	Europe and Asia	Prospective clinical trial	2023	NR	40	8.5	NR
Geavlete B. et al. [13]	Romania	Retrospective study	2022	Yes	59	48.94 ± 15.93	3 months
Soundarya G. et al. [14]	India	Prospective clinical trial	2022	NR	52	40.91 ± 12.62	5.98 ± 0.79 months
Sytnik D. et al. [15]	Russia	Prospective clinical trial	2022	Yes	12	NR	
Taraktin M. et al. [4]	Russia	Prospective Clinical study	2022	Yes	153	54 ± 2.8	3 months
Vaddi C.M. et al. [16]	US	Prospective study	2022	Yes	126	45.04 ± 12.30	3 months
Sierra et al. [17]	Japan	Prospective study	2021	Yes	50	55(44–61.5)	NR
Taraktin M. et al. [18]	Russia	Retrospective study	2021	NR	14	NR	3 months
Enikeev D. et al. [19]	Russia	Prospective study	2020	Yes	40	56	3 months
Korolev D. et al. [20]	Russia	Clinical study	2020	NR	130	NR	NR

#### Outcome measurement of included studies

The studies exhibit diversity regarding stone characteristics, encompassing volume, size, and density variations. They also span a range of outcomes, including mean operative times, laser on time, stone-free rates, ablation efficacy, ablation speed, and complications. Stone-free rates demonstrated variability, with several studies achieving notably high success rates. These rates ranged from 72 to 100%. Stone density, measured in Hounsfield Units (HU), spans from 250 to 1610 HU, showcasing the varied composition of stones and their potential impact on treatment response.

The mean operative times range from 5.4 to 25.9 min, reflecting the procedural complexity and potential differences in stone fragmentation approaches. The highest reported laser on time (LOT) recorded was 56 min, which suggests notable variations in the duration of laser application throughout the procedural course. Ablation efficacy, measured in J/mm3, ranges from 1.7 to 13.3, representing the efficiency of laser energy in breaking down stones.

Ablation speed, expressed in mm3/sec, ranges from 0.3 to 1.7 for ureteral stones and from 0.4 to 3.9 for renal stones, indicating the rate at which stones disintegrate during the procedure.

The spectrum of reported complications was equally diverse, extending from no complications to an overall postoperative complication rate of 7.6%. The reported complications varied across studies, mainly featuring Clavien grade I–II complications, while a subset encountered more severe Clavien grade III–V complications. Additionally, other studies documented various complications, such as haematuria, fever, transient creatinine elevation, and various postoperative challenges like bleeding, pain, and sepsis. Overall, the mean length of hospital stay was 3 days as reported in only 2 of the included studies (Tables 3 and 4).

**Table 3:** Summary of the outcomes of the studies included in this review

	**Stone Characteristics**			**Outcomes**					**Complication**
	**Stone Volume**	**Stone size (mm)**	**Density (HU)**	**Mean operative times**	**Laser on time (LOT)**	**Stone free rate**	**Ablation efficacy (J/mm3)**	**Ablation speed (mm**^**3**^**/sec)**	
Delbarre et al. [10]	NR	20.4 (17.3)	NR	62 (25.5)		72%	NR	NR	Clavien grade I–II (9), Clavien grade III–V (4)
Singh A et al. [11]	1753.12 ± 1245.81 (1169.27–2193.25) mm^3^		1104.46 ± 313.09 (875.00–1317.00) HU	43.38 ± 12.96 (35.00–51.25) min	537.79 ± 689.89 (21.00–1080.00) sec	NR	20.30 ± 15.5 (8.88–25.57) J/mm^3^	1.32 ± 0.7 (0.82–1.64) mm^3^/sec	Clavien grades 1–2
Quiroz Madarriaga Y. et al. [12]	NR	9.7 mm (4–19 mm)	NR	55 min	NR	90%	NR	NR	No complications
Geavlete B. et al. [13]	NR	13.25 ± 4.74	1045.1 ± 109.19	64.06 ± 15.01	Nil	96.61%	NR	NR	Haematuria, fever
Soundarya G. et al. [14]	NR	18.34 ± 3.06 mm	1014.06 ± 244.82 HU	NR	1242.69 ± 515.20 s	88.40%	Mean laser efficacy:9.37 ± 4.17	1.80 ± 0.63 mm3 /sec	No complication more than Grade 2 occurred, hematuria, fever, and transient rise in serum creatinine
Sytnik D. et al. [15]	NR	on the right—12 mm ± 2.6 mm; on the left—11.7 mm ± 2.4 mm)	1134 ± 217 HU	56 ± 11	Nil	83.3%	NR	NR	Increased body temperature (one patient) (Clavien-Dindo II)
Taraktin M. et al. [4]	279.6(139.4–615.8)	12.5 ± 8.8 (7–30)	1020 ± 382(250–1900)	NR	2.7(1.6–6.6)	89%	13.3 (7.3–20.9)	1.7 (1.0–2.8)	Fever, Transient creatinine elevation, Urinary tract infection
Vaddi C.M. et al. [16]	1061.85 ± 806.81 mm3	15.19 ± 4.52	985.82 ± 302.57	NR	19.78 ± 12.32	93.60%	Mean laser efficacy: 14.35 ± 5.70	0.86 ± 0.31	Haematuria, fever, Clavien grades 1–2
Sierra et al. [17]	ureteral: 486 (332–1250) mm^3^, renal: 1800 (682.8–2760) mm^3^	ureteral: 347 (147–1800) mm3 renal:1125 (294–4000) mm3	ureteral stones 998 (776–1300) HU; renal stones 1200 (750–1300) HU	NR	23 (14.2–38.7)	100%	ureteral;8.7 (4.8–65.2); renal:14.3 (7.8–24.7)	ureteral:0.3 (0.2–1.3); renal:0.7 (0.4–1.2)	Clavien grades 2
Taraktin M. et al. [18]	2743 (1451–4213)	23.5 ± 6.3 (20–36)	833.8 ± 298.3 (400–1394)	NR	11.7 (10.0–15.5)	85.70%	4.6 (2.9–5.5)	3.9 (3.9–5.7)	Fever, Transient creatine elevation
Enikeev D. et al. [19]	883 (606–1664) mm3	883 (IQR 606–1664) mm3	880 ± 381 HU	23.1 ± 10.9	G1: 0.5 J × 30 Hz 15 W; G2: 372 (96–414)	92.50%	G1:2.7 (1.8–9.8); G2:4.8 (2.6–11.3)	G1:5.5 (1.5–8.7) G2:8.5 (3.6–19.0)	Clavien 1–2
Korolev D. et al. [20]	1^st^ stone:1,531.8 (5,592.2); 2^nd^ stone: 690.4 (1,606.6); 3^rd^ stone: 561.1 (977.4)		900.7 (370.6), 862.0 (373.7), 634.1 (399.6)	59.4 ± 31.5	NR	NR	NR	NR	Postoperative bleeding (1), fever (1), pain (2), sepsis (3), stent loss (1)

**Table 4:** Summary of outcomes in terms of hospital stay and impact on harder stones

	**Length of stay**	**Effect on harder stones**
Delbarre et al. [10]	NR	NR
Singh A et al. [11]	NR	Improved stone volumes (> 1000 mm3) result in improved laser efficiency since each mm3 of stone requires less energy to be removed
Quiroz Madarriaga Y. et al. [12]	NR	NR
Geavlete B. et al. [13]	NR	NR
Soundarya G. et al. [14]	NR	Altogether, 88.4% of the population was stone-free. The average energy utilized was 18,508.81 ± 8356.60 Joules. While laser effectiveness on average was 9.37 ± 4.17 J/mm3. The average rate of ablation was 1.80 ± 0.63 mm3/sec
Sytnik D. et al. [15]	NR	NR
Taraktin M. et al. [4]	Mean: 3 days	There were 87 (56.2%) patients with higher-density stones (> 1,000 HU). The median for laser on time was 2.9 (1.7–5.6) minutes. The median total energy for stone ablation was 3.9 (2.3–6.8) kJ, the median ablation speed was 1.3 (0.9–2.5) mm3/s, the median ablation efficacy was 16.2 (8.6–22.8) J/mm3, and the energy consumption was 160.1 (64.8–593.5) J/s. The mean stone density was 1,305.9 ± 194.1 (1,000–1,900) HU
Vaddi C.M. et al. [16]	NR	With a mean laser time of 19.78 ± 12.32 min. Higher stone volume (> 1000 mm3) resulted in a substantial decrease in J/mm3 from 16.18 ± 5.90 to 10.92 ± 3.21 (*P* < 0.001) and a considerable improvement in ablation speed
Sierra et al. [17]	NR	NR
Taraktin M. et al. [18]	Mean 3–4 days in RIRS and PCNL group	NR
Enikeev D. et al. [19]	NR	NR
Korolev D. et al. [20]	NR	NR

*NR* not reported, *PCNL* percutaneous nephrolithotomy

**Fig. 2:**
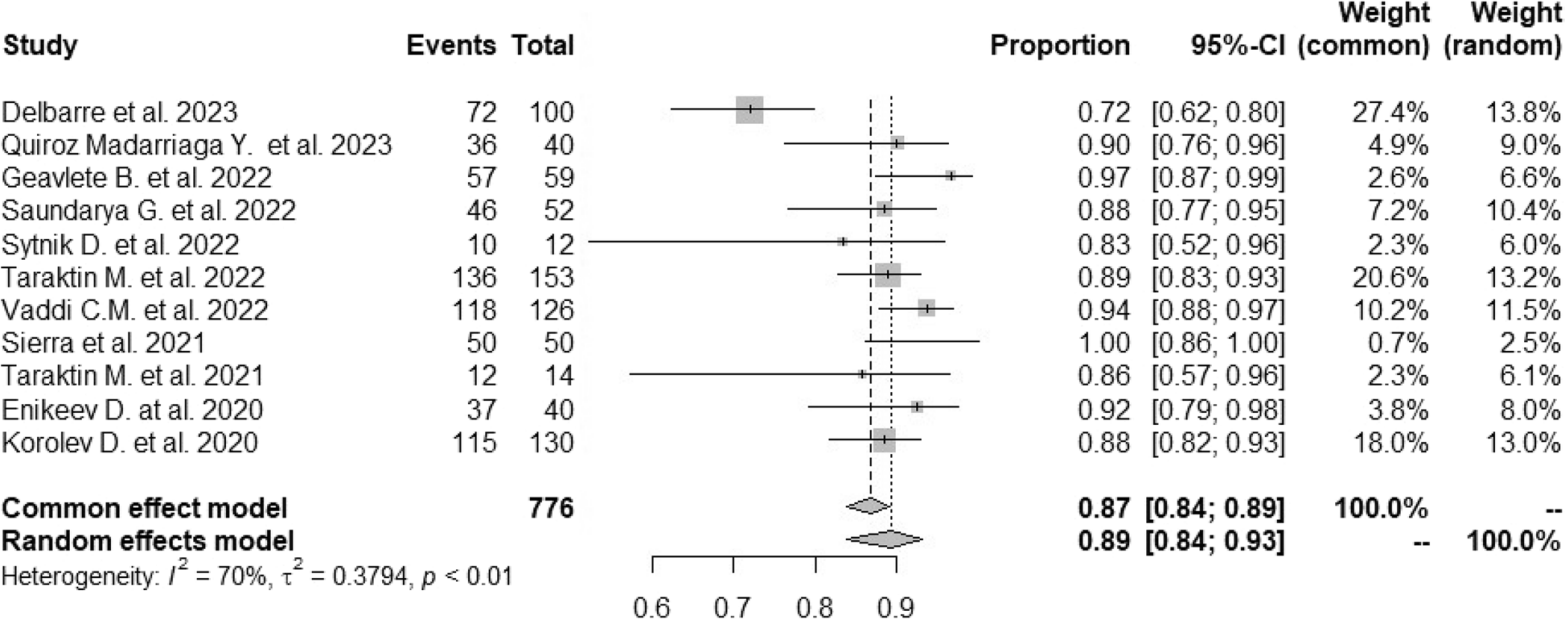
Forest plot showing the stone-free rate of included studies

A predicted proportion of the stone-free rate of 89.37% (95% CI: 83.93% to 93.12%) suggests that, on average, approximately 89.37% of patients achieved a stone-free state after treatment (Fig. 2). Significant heterogeneity exists among the studies, as indicated by a Q-value of 33.1174 and a corresponding *p*-value of 0.0003. This implies that the observed differences in effect sizes between the studies are unlikely to be due to chance alone.

The I^2^ value of 69.80% (95% CI: 25.91% to 92.02%) reflects the proportion of total variability in the observed effect sizes attributed to true heterogeneity among the studies rather than sampling error. This suggests that a substantial proportion of the observed variability is due to differences between the studies.

The H^2^ value of 3.31 (95% CI: 1.35 to 12.53) indicates that the total variability observed is approximately 3.31 times larger than what would be expected due to sampling variability alone. This suggests evidence of heterogeneity beyond what could be explained by chance alone.

Additionally, the model results show that the estimated overall effect size (logit-transformed) is 2.1289, with a standard error of 0.2429. The associated z-value of 8.7648 indicates that the effect size is highly statistically significant (*p* < 0.0001), further supporting the significance of the overall effect. The confidence interval for the effect size is 1.6528 to 2.6049.

The analysis indicates a high stone-free rate with moderate to high heterogeneity among the studies. The Q-test, I^2^, and H^2^ values provide insight into the extent of heterogeneity and its contribution to the observed variability in effect sizes. The statistically significant overall effect size suggests a consistent trend among the studies.

## Publication bias assessment

**Fig. 3:**
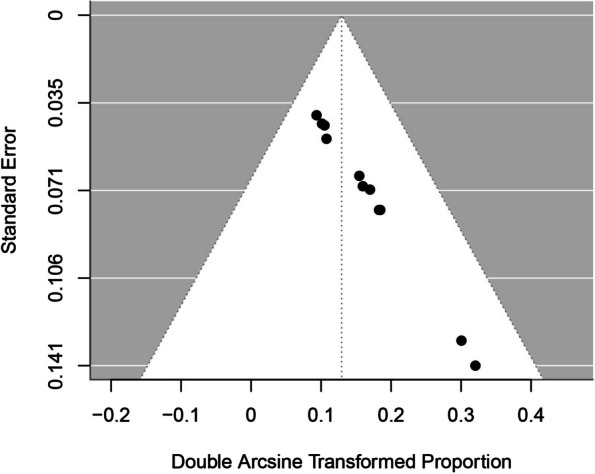
Funnel Plot for Stone-Free Rate in the Meta-Analysis (Publication Bias Assessment)

The results of this analysis suggest that there is statistically significant funnel plot asymmetry, indicating the presence of potential publication bias or small-study effects in the meta-analysis. The limit estimate of the coefficient as the standard error approaches zero is near zero (-0.0001), and the confidence interval is quite wide (-0.1035 to 0.1033), which suggests a lack of a strong effect as SE decreases but with considerable uncertainty (Fig. 3).

## Discussion

With higher absorption coefficients, a shorter beam profile, and the ability to reach low pulse energies and high pulse frequencies, in vitro studies suggest that the TFL is superior to the Ho: YAG laser for lithotripsy. These factors result in higher ablation rates, less retropulsion, less fiber tip degradation, and less dust generation. While most of the theoretical benefits of the TFL have been demonstrated in clinical settings, the factors influencing these outcomes still need to be discovered because the TFL is a new technology with few studies currently available. However, TFL has demonstrated a reasonable stone-free rate and low complication rates [21]. A super-pulsed TFL with a wavelength of 1.94 m and a maximum power output of 40 W has exhibited greater efficiency in vitro compared to Ho: YAG while having the same safety profile, as per the currently available literature. The most efficient way to do stone cleaning during RIRS is to utilize a TFL with a pulse energy of 0.025–6 J and a high repetition rate of up to 1600 Hz. When performing RIRS, a super pulsed TFL with a wavelength of 1.94 m and a maximum power of 500 W has demonstrated high efficiency because it enables good endoscopic imaging, minimal retropulsion, and stone dusting, all of which have a positive impact on the stone-free rate [5].

The findings of this study demonstrated that, on average, approximately 89.37% of patients achieved a stone-free state after treatment. While comparing the stone-free rate with Ho: YAG laser, Aboumarzouk et al. reported that almost 87.7% achieved stone-free status. There were no significant complications; only 11% of the patients experienced mild complications, with only 4% experiencing minor bleeding. The average stone size ranged from 5–35 mm, with a mean of 13.2 mm [22]. Study findings by Farkas et al. depicted that the direct success rates in the upper, middle, and lower ureters were 84.6%, 88.7%, and 94.8%, respectively, implying that the ureters were free of stones on the first postoperative day. The final success rates were 84.6%, 96.7%, and 96.7%, respectively, indicating stone-free ureters four weeks following the operation without a second intervention [23]. Both of these studies exhibited slightly lower stone-free rates than our results; however, in the Farkas et al. study, the stone-free rate for the middle and lower ureters was comparatively higher than our reported percentage.

Results of a multicentric survey among children revealed a slightly higher stone-free rate than ours since they reported that 97.3% of the study population were stone-free overall through a Ho: YAG laser. Four remaining stone fragments that were effectively treated using the same method and two incidences of stent migration made up the 4.0% postoperative complications rate overall. The re-operation rate was strongly influenced by the position of the proximal stone and the presence of residual fragments larger than 2 mm on multivariate analysis. In comparison, 10.3 mm was the average stone size (with a 5–17 range) [24]. However, results of a randomized control trial concluded that compared to Ho: YAG, TFL considerably increased the percentage of renal stone patients who were stone-free and reduced intraoperative complications. Hence, for stone lithotripsy, TFL is becoming the preferred laser procedure [25]. Similarly, due to its greater single-stage stone-free rate, TFL was preferred by Castellani et al. over Ho: YAG laser with MOSES technology in flexible ureteroscopy for renal stones [26].

Understanding super-pulse TFL effectiveness and safety primarily relies on its comparative analysis with the Ho: YAG laser, which is currently considered the gold standard for laser lithotripsy. TFL machine characteristics include electronically controlled laser diodes that provide constant peak power of up to 500W with the same pulse energy range as any high-power Ho: YAG laser. One advantage of the TFL over the Ho: YAG laser is its frequency capabilities, as it can achieve frequencies of over 2000 Hz, compared to the Ho: YAG laser's maximum frequency of 120 Hz. Additionally, the TFL can be operated with very low pulse energies and very long pulse durations of up to 50 ms, which is impossible with the Ho: YAG laser and gives the TFL a significant advantage. TFL offers pulse frequencies up to 2400 Hz and features long pulse durations of up to 50 ms.

Moreover, TFL is significantly smaller and lighter than Ho: YAG lasers and operates quietly due to its air-cooling mechanism while consuming less energy. TFL has been shown to fragment twice as fast as Ho: YAG and dust four to five times faster than Ho: YAG, potentially leading to less operating room time. In addition, retropulsion is less frequent than with Ho: YAG, with clinically significant retropulsion observed at 1J compared to 0.2J with Ho: YAG [27]. Findings of a randomized clinical trial showed that the Ho: YAG group required postoperative stenting in one vs. four cases, and the entire operation and lasering times were higher (24.7 0.7 vs. 32.4 0.7 min, *p* = 0.05). After 30 days of monitoring, the super-pulsed TFL group had no residual stones compared to five Ho: YAG group incidences. Therefore, the super-pulsed TFL technique is considered to have a tremendous efficacy-to-safety ratio. As a potential substitute for Ho: YAG laser stone management, the super-pulsed TFL may be considered [28]. Similarly, Carrera et al. concluded that high-power super pulse TFL proved to be a potential new technology for treating urolithiasis by effectively ablating a wide range of stone types while maintaining a safety profile equivalent to the currently recognized gold standard approaches [29].

In this study, the impact of super pulse TFL RIRS specifically on harder stones was also evaluated. Four studies among the included studies reported the effects and outcomes of RIRS on harder stones > 1000 mm3 or HU. Findings of a study by Singh et al. noted that Improved stone volumes (> 1000 mm3) resulted in improved laser efficiency since each mm3 of stone requires less energy to be removed [11]. Similarly, results of a study by Vaddi et al. also stated that Higher stone volume (> 1000 mm3) resulted in a substantial decrease in J/mm3 from 16.18 ± 5.90 to 10.92 ± 3.21 (*P* < 0.001) and a considerable improvement in ablation speed [16]. While Tarakatin et al. observed that for patients with higher-density stones (> 1,000 HU). The median for laser on time was 2.9 (1.7–5.6) minutes [4]. Soundraya et al. reported that 88.4% was the stone-free rate achieved for the harder stone population, and the average energy utilized was 18508.81 ± 8356.60 Joules. While laser effectiveness on average was 9.37 ± 4.17 J/mm3 [14]. These findings overall suggest the positive impact of super pulse TFL RIRS even on harder stones. However, these findings could not be compared elaborately with other studies due to the dearth and scarcity of studies in literature in this regard.

Furthermore, given that the wavelength of water's absorption peak is approximately 1,940 nm, one of super pulse TFL's primary benefits is that it has a wavelength that is exceptionally close to this peak. Hence, it is anticipated that the TFL can be up to 4–5 times more effective in stone ablation than the Ho: YAG laser in identical circumstances, just based on the wavelength consideration. Its effectiveness during RIRS may be facilitated by prior preclinical investigations that showed super-pulsed TFL to have up to 4 times greater ablation efficacy and a considerable reduction in retropulsion [30–33]. Andreeva et al. further commented that in comparison to a Ho: YAG laser, the superpulse TFL has shown to have ablation rates up to three times higher, a retropulsion effect that is three times lower, and a burn back factor that is more than two times lower. The superpulse TFL provides a flexible laser platform that implements the ideal lithotripsy parameters [31]. Moreover, Taratkin et al. highlighted the importance of super-pulsed TFL in RIRS by concluding that regardless of the nature and density of the stone, super-pulsed TFL is a reliable and effective tool in lithotripsy. Super-pulsed TFL maintains visibility while minimizing the retropulsion [30].

Corrales and Traxer concluded that TFL has a low complication rate and is considered a safe and effective technique for lithotripsy during RIRS, as results demonstrated that none of the complications were caused by TFL, signifying its safety and reproducibility among patients [34]. However, our findings have demonstrated a diverse spectrum in this aspect, which ranged from no complications noted to a post-procedural complication rate of 7.6%. Most of the included studies reported haematuria, a transient rise in creatinine, and fever, along with other studies reporting Clavien grade 1 and 2 complications, while Delbarre et al. reported grade 3 and 4 complications [10]. Additionally, Korolev et al. [20] reported postoperative bleeding and sepsis [20]. This trend still suggests super pulse TFL as a safe and effective procedure since only two studies reported significant complications that were also managed effectively. Liu et al. also concluded that utilizing a super pulse TFL to treat urinary calculi is practical and safe [35]. Compared to the Ho: YAG laser, the results of a study by Sonmez et al. depicted that the laser-setting parameters exhibited significant fluctuation as the stone size and Hounsfield unit values rose. Longer anesthesia, time of surgery, hospital stays, and a higher risk of local trauma with a post-ureteroscopic lesion scale grade were associated with increased laser setup parameters [36]. However, in our study with super pulse TFL, the operating time range was reported from 5.4 to 25.9 min, and the mean length of stay was three days. Our study is one of the few available meta-analyses and systemic reviews assessing the outcomes of super pulse TFL exclusively, to the best of our knowledge. This study's main advantages and strengths are the systematic search methodology and the analysis of all keywords in this field. However, we could not elaborately compare our findings to another exclusive super pulse TFL since, to date, there needs to be more data on the clinical outcomes of TFL as it is a newly available technology worldwide. Therefore, we recommend further comparative and prospective randomized controlled trials and long-term follow-up studies to confirm the benefits of TFL.

## Limitations

The study has several limitations that warrant consideration. Firstly, the predominantly varying age range of patients, most under 70 years old, may restrict the generalizability of findings to older populations. Additionally, the need for standardized criteria for determining stone-free rates among the included studies introduces uncertainty in assessing treatment efficacy. The potential influence of confounding factors, such as patients' comorbidity status, must be addressed extensively, which could impact treatment outcomes and conclusions. Variability in surgical expertise among operators performing RIRS with TFL may have influenced treatment outcomes and complication rates. Most studies' relatively short follow-up periods (around three months) might not capture long-term complications or stone recurrence rates. Our meta-analysis did not include direct comparisons with other groups or control groups, limiting the ability to make direct comparisons and attribute outcomes solely to TFL treatment. Variations in stone characteristics, including volume, size, and density, among studies could affect TFL's fragmentation efficacy and stone-free rates. Moreover, including studies across different regions and patient populations introduces ethnic and geographic variability that could influence treatment responses.

The analysis has unveiled a noteworthy high average stone-free rate; however, it is important to acknowledge the presence of moderate to high heterogeneity among the studies. Stone-free rate definitions varied among the studies. These variations in stone-free rate definitions are crucial when interpreting our findings and assessing the heterogeneity in the results. This heterogeneity is evident in the differences in stone characteristics, surgical outcomes, and complications among the included studies. We have quantified this heterogeneity through various statistical measures, including the Q-test, I^2^, and H^2^ values, illuminating its influence on the observed variability in effect sizes. Despite this heterogeneity, the persistence of a statistically significant overall effect size underscores a consistent trend across the studies, emphasizing the treatment's effectiveness. It is vital for both researchers and readers to remain mindful of this heterogeneity and its potential impact when interpreting the study's findings and considering their generalizability.

## Conclusion

This meta-analysis suggests that RIRS using a super-pulsed thulium fiber laser is an effective and safe treatment option for stones, with high stone-free and low complication rates. The included studies exhibited a low risk of bias and were of high quality. However, more extensive randomized controlled trials with extended follow-up periods are needed to investigate this technique's efficacy and safety.

## Data Availability

The data supporting this study's findings are available from the corresponding author upon reasonable request.
